# Vangl2 Promotes Hematopoietic Stem Cell Expansion

**DOI:** 10.3389/fcell.2022.760248

**Published:** 2022-03-24

**Authors:** Sarah Bouali, Roxann Hétu-Arbour, Célia Gardet, Krista M. Heinonen

**Affiliations:** ^1^ Institut National de La Recherche Scientifique, INRS-Centre Armand-Frappier Santé Biotechnologie, Université du Québec, Laval, QC, Canada; ^2^ Institut des Sciences et Industries du Vivant et de l’Environnement - AgroParisTech, Université Paris-Saclay, Paris, France

**Keywords:** hematopoietic stem cells, Vangl2, wnt/planar cell polarity pathway, bone marrow transplants, hematopoietic recovery

## Abstract

Regulation of hematopoietic stem cell (HSC) self-renewal and differentiation is essential for their maintenance, and HSC polarity has been shown to play an important role in this regulation. Vangl2, a key component of the Wnt/polarity pathway, is expressed by fetal and adult HSCs, but its role in hematopoiesis and HSC function is unknown. Here we show the deletion of Vangl2 in mouse hematopoietic cells impairs HSC expansion and hematopoietic recovery post-transplant. Old *Vangl2-*deficient mice showed increased expansion of myeloid-biased multipotent progenitor cells concomitant with splenomegaly. Moreover, *Vangl2*-deficient cells were not able to effectively reconstitute the recipient bone marrow in serial transplants, or when coming from slightly older donors, demonstrating impaired self-renewal or expansion. Aged *Vangl2*-deficient HSCs displayed increased levels of cell cycle inhibitor p16^INK4a^ and active β–catenin, which could contribute to their impaired function. Overall, our findings identify Vangl2 as a new regulator of hematopoiesis.

## Introduction

Hematopoiesis serves to generate trillions of new blood cells every day from a small number of hematopoietic stem cells (HSC). HSCs possess the ability to self-renew and differentiate into erythroid, myeloid and lymphoid cells. This is clinically used in bone marrow transplantation (BMT), in which non-functional HSCs are replaced with healthy ones. BMT is the most effective cell therapy used to treat hematopoietic malignancies, especially blood cancers. Healthy HSCs from compatible donors are traditionally obtained from bone marrow (BM), but can also be obtained from cytokine-mobilized peripheral blood or umbilical cord blood. Understanding HSC regulators could help studying HSCs *ex vivo* which remains a prominent challenge today and improve BMT success by expanding the use of cord blood HSCs which are associated with fewer risks of graft-versus-host-disease (Li and Sykes, 2012).

HSCs reside in the BM niche, where they receive different cues regulating their survival and quiescence vs. proliferation. Among these, Wnt proteins (Staal et al., 2008) regulate the balance between HSC proliferation and maintenance through three interconnected pathways: the canonical Wnt/β-catenin pathway, extensively studied and known to control HSC self-renewal (Luis et al., 2012), and non-canonical Wnt/Ca^2+^ (Florian et al., 2012; Florian et al., 2013) and Wnt/Planar Cell Polarity (PCP) (Le Grand et al., 2009; Sugimura et al., 2012; Abidin et al., 2015; Lhoumeau et al., 2016) pathways, mostly known for stem cell maintenance. Wnt/PCP pathway was originally identified as a means to control epithelial cell polarity (Seifert and Mlodzik, 2007), but its role in HSC regulation has been more recently discovered. Previous work from our laboratory suggests a protective role for Wnt/PCP signaling in HSCs, particularly under hematopoietic stress (Heinonen et al., 2011; Abidin et al., 2015). Frizzled-6, a receptor which has a prevalent role in non-canonical Wnt/PCP signalling (Chang et al., 2016), regulates HSC self-renewal and progenitor cell survival post-transplant and is crucial for emergency hematopoietic response during inflammation (Abidin et al., 2015) and infections (Abidin et al., 2017). Others have demonstrated that mice deficient in the Wnt/PCP component Ptk-7 exhibited deregulated HSC proliferation and migration (Lhoumeau et al., 2016), while Frizzled-8 and Celsr2/Fmi maintained HSC long-term quiescence in the BM (Sugimura et al., 2012). In sum, various Wnt/PCP pathway components have been implicated in regulating HSC proliferation and maintenance.

Vangl2 is another key regulator of the Wnt/PCP signalling pathway, and fetal and adult HSCs express Vangl2 (Kwarteng et al., 2018). Moreover, Vangl2 is expressed more strongly by HSCs and progenitor cells compared to mature blood cells (Choi et al., 2019). Vangl2 is known for its crucial role in neural plate development, and mutations in this gene are associated with neural tube defects in mice (Murdoch et al., 2001) and in humans (Kibar et al., 2011). In mouse epithelial cells, Vangl2 is known to control hair bundle orientation and plays a role in Frizzled-3 and Frizzled-6 cell surface localisation in the inner ear (Montcouquiol et al., 2003; Wang et al., 2006). Despite being highly expressed by HSCs and progenitor cells and having a crucial role in the PCP pathway, nothing is known yet about the role of Vangl2 in HSC regulation and maintenance.

In this study, we show that the loss of Vangl2 in hematopoietic cells impairs post-transplant recovery and leads to BM failure following serial transplantation. There are also alterations in the multipotent progenitor cell (MPP) pool of old *Vangl2*-deficient mice, and old *Vangl2*-deficient HSCs show increased expression of the senescence-associated cell cycle inhibitor p16^INK4a^ concomitant with increased β-catenin stabilization. These results uncover the important role of Vangl2 in HSC expansion and post-transplant recovery and open the door for further research on Vangl2 as a regulator of hematopoiesis and HSC function.

## Materials and Methods

### Experimental Animals

C57BL/6, B6.SJL (B6.SJL-*Ptprc* ^*a*^ *Pepc* ^*b*^/BoyJ), B6.Cg-*Commd10* ^*Tg(Vav1-icre)A2Kio*^/J (Vav-Cre), and B6;129-*Vangl2* ^*tm2.1Mdea*^/J (*Vangl2* ^lox/lox^) mice were purchased from The Jackson Laboratory (Bar Harbor, ME). Male Vav-Cre^+^ *Vangl2* ^lox/lox^ or Vav-Cre^+^ *Vangl2* ^lox/+^ mice were crossed with *Vangl2* ^lox/+^ or *Vangl2* ^lox/lox^ females to obtain offspring in which the Vangl2 gene is deleted from the hematopoietic stem/progenitor cell pool (Vav-Cre^+^ *Vangl2* ^lox/lox^). Experiments were done using both male and female mice together with sex-matched, co-housed littermate controls (Vav-Cre^-^ *Vangl2* ^lox/+^ or Vav-Cre^-^ *Vangl2* ^lox/lox^). All mice were reared and housed in pathogen-free conditions in sterile ventilated supports at the animal facility of l’Institut national de la recherche scientifique (INRS) - Centre Armand-Frappier Santé Biotechnologie (*Laboratoire national de biologie expérimentale*). All procedures were carried out in accordance with the Canadian Council on Animal Care guidelines and were approved by INRS Institutional Animal Care Committee (CIPA).

### BM Transplantation Assays

Donor (CD45.2^+^; C57BL/6 background, control and *Vangl2* ^Δ/Δ^) and competitor (CD45.1^+^; B6.SJL) BM cells were analyzed by flow cytometry prior to transplant, and the quantities of total BM cells were adjusted to inject equivalent numbers of donor and competitor HSCs, similar to what has been previously described and as detailed below (Kwarteng and Heinonen, 2016; Hétu-Arbour et al., 2021a). Cells were pooled from two to three donors per experiment, and quantities of whole BM cells were normalized to the equivalent of 150 LT-HSCs (defined as CD150^+^ CD48^−^ Lin^−^ Sca1^+^ c-Kit^hi^). This corresponded to approx. 1 × 10^6^ CD45.1^+^ competitor BM cells and, on average, to 7 × 10^5^ total BM cells for CD45.2^+^ donors. The exact number was determined independently for each donor based on LT-HSC frequency in the BM. Cells were injected into the lateral tail vein of lethally irradiated recipient mice (CD45.1^+^ CD45.2^+^; F1 offspring of C57BL/6 x B6.SJL intercrosses). Recipients were given two doses of 4.5 Gy with a 16-h interval using the RS 2000 small animal X-ray irradiator (RadSource Technologies, Suwanee, GA). For secondary transplants, an equal number (2.5 × 10^6^) of total BM cells from two primary recipients were pooled, mixed with 1 × 10^6^ fresh CD45.1^+^ competitor cells, and injected into lethally irradiated secondary recipients. For tertiary transplants, BM cells were again pooled from two secondary recipients (5 × 10^6^ in total), but there was no added competition. Serial transplant results are shown for young primary donors, only. To analyze short and long-term reconstitution, peripheral blood was collected from the mandibular vein of recipient mice at 4, 8, 12, and 16 weeks post-transplant. Mice were euthanized 20–22 weeks post-transplant and BM/spleen were collected and prepared as described below for flow cytometry analysis.

### Flow Cytometry and Imaging

BM cells were harvested by flushing tibiae and femora with PBS/0.1% BSA/0.5 mM EDTA using a syringe with a 25-gauge needle. Spleen cells were isolated by mechanically crushing the organs with the plunger of a 10cc syringe. For blood analysis, erythrocytes were lysed in hypotonic buffer (0.14M NH_4_Cl; 17 mM Tris-HCl, pH 7.6) for 4–6 min, or until sample was translucent and reaction was stopped with three volumes of ice-cold PBS, followed by centrifugation and washing with PBS/0.1% BSA/0.5 mM EDTA. Non-specific staining was blocked with anti-CD16/CD32 prior to staining with fluorochrome-conjugated antibodies for 30 min on ice. For cell cycle analysis, cells were first stained with surface antibodies, then fixed and permeabilized using the Foxp3 labeling kit for nuclear proteins as directed by the manufacturer (eBioscience, San Diego, CA). Samples were blocked with 2% rat serum for 30 min before intracellular staining with anti-Ki-67 for 1 h, followed by DAPI (0.25 μg/ml) for 30 min, both at room temperature. For detection of p16 expression, cells were prepared as described above, fixed, permeabilized, and then blocked with 1% BSA before intracellular staining with the recombinant anti-CDKN2A/p16^INK4a^ antibody for 1 h at room temperature. Primary antibody was detected with Alexa Fluor 488-conjugated F(ab')2-goat anti-rabbit IgG (H + L) cross-adsorbed secondary antibody. Samples were acquired on a four-laser BD LSRFortessa flow cytometer (BD Biosciences, Mountain View, CA) and analyzed using FACS DiVa software (v. 8.1) or FlowJo (v. 10.1) software. See Table 1 for additional details on antibodies.

**TABLE 1:** List of antibodies used for flow cytometry.

Analysis	Antibody name	Clone	Fluorochrome	Dilution	Company
**BM HSCs and progenitor cells**	CD16/32 (Fc block)	2.4G2	Purified	1/100	BD Biosciences
	CD48	HM48-1	BV421	1/400	BD Biosciences
	CD117 (c-kit)	2B8	PE	1/400	BD Biosciences
	Ly-6A/E (Sca1)	D7	PE-Cy7	1/800	BD Biosciences
	CD150 (SLAM)	TC15-12F12.2	Alexa Fluor 647	1/400	BioLegend
	CD11b	M1/70	biotin	1/400	BD Biosciences
	Ter119	TER-119	biotin	1/800	BD Biosciences
	CD3ε	145-2C11	biotin	1/400	BD Biosciences
	B220 (CD45R)	RA3-6B2	biotin	1/400	BD Biosciences
	Gr1 (Ly-6C/G)	RB6-8C5	biotin	1/400	BD Biosciences
	CD135 (Flt3)	A2F10	PerCP-eFluor 710	1/400	eBioscience
	CD45.1	A20	APC-Cy7	1/100	BD Biosciences
	CD45.2	104	FITC	1/200	BD Biosciences
	Streptavidin	561419	V500	1/1600	BD Biosciences
**Peripheral blood**	CD45.2	104	FITC	1/200	BD Biosciences
	CD19	1D3	PE	1/800	BD Biosciences
	CD3ε	145-2C11	PE-Cy7	1/400	BD Biosciences
	CD45.1	A20	APC	1/200	eBioscience
	Gr1 (Ly-6C/G)	RB6-8C5	APC-Cy7	1/800	BD Biosciences
**Mature BM/Spleen cells**	CD11c	HL3	bv711	1/800	BD Biosciences
	CD19	1D3	PE	1/800	BD Biosciences
	CD3ε	145-2C11	PE-Cy7	1/400	BD Biosciences
	CD11b	M1/70	APC	1/800	BD Biosciences
	IgD	11-26c.2a	APC	1/800	BD Biosciences
	Gr1 (Ly-6C/G)	RB6-8C5	APC-Cy7	1/800	BD Biosciences
	CD4	GK1.5	APC-Cy7	1/400	BD Biosciences
	CD8a	53-6.7	V450	1/800	BD Biosciences
	CD45.1	A20	FITC	1/200	eBioscience
**Cell cycle**	CD48	HM48-1	PerCPCy5.5	1/400	eBioscience
	CD117 (c-kit)	2B8	PE	1/400	BD Biosciences
	Ly-6A/E (Sca1)	D7	PE-Cy7	1/800	BD Biosciences
	CD150 (SLAM)	TC15-12F12.2	Alexa Fluor 647	1/400	BioLegend
	CD11b	M1/70	biotin	1/400	BD Biosciences
	Ter119	TER-119	biotin	1/800	BD Biosciences
	CD3ε	145-2C11	biotin	1/400	BD Biosciences
	B220 (CD45R)	RA3-6B2	biotin	1/400	BD Biosciences
	Gr1 (Ly-6C/G)	RB6-8C5	biotin	1/400	BD Biosciences
	Streptavidin	563262	BV711	1/400	BD Biosciences
**Imaging flow cytometry**	CD117	2B8	BB515	1/800	BD Biosciences
	Ly-6A/E (Sca1)	D7	PE-Cy7	1/400	BD Biosciences
	CD150 (SLAM)	TC15-12F12.2	Alexa Fluor 647	1/400	BioLegend
**Intracellular staining**	Foxp3/Transcription Factor Set	-	-	-	eBioscience (cat# 00-5523-00)
	KI-67	SolA15	FITC	1/200	eBioscience
	DAPI	-	Purified	0.25 µg/mL	Life Technologies (cat# D3571)
	Cdc42	EPR15620	Purified	1/170	Abcam
	Non-phospho active β-catenin	D13A1	Purified	1/200	Cell Signalling Technology
	F(ab')₂ Fragment Goat Anti-Rabbit IgG (H+L)	-	R-PE	1:1500 (Cdc42), 1:500 (β-catenin)	Jackson Immunoresearch Labs (cat# 111-116-144)
	CDKN2A/p16^INK4a^	EPR20418	Purified	1/200	Abcam
	F(ab')2-Goat anti-Rabbit IgG (H+L)	-	Alexa Fluor 488	1/8000	Invitrogen (cat# A11070)
**Colony assays**	CD117 (c-kit)	2B8	PE	1/400	BD Biosciences
	CD11c	HL3	PECy7	1/800	BD Biosciences
	CD11b	M1/70	APC	1/800	BD Biosciences
	Gr1 (Ly-6C/G)	RB6-8C5	APC-Cy7	1/800	BD Biosciences

For imaging flow cytometry, BM cells were harvested by flushing tibiae, femora and iliac crests with PBS using a 25-gauge syringe needle. Samples were first enriched for HSCs and progenitor cells with the EasySep™ Mouse Hematopoietic Cell Isolation Kit (Stem Cell Technologies, Vancouver, BC, Canada) and then stained with anti-Sca1, anti-CD117 and anti-CD150 (see Table 1). Cells were washed with PBS, fixed, permeabilized and blocked with BSA as described above prior to intracellular labeling with anti-Cdc42 or anti-non-phospho (active) β‐catenin overnight. Intracellular staining was detected with R-Phycoerythrin-conjugated AffiniPure F(ab')₂ Fragment Goat Anti-Rabbit IgG (H + L) for 1 h and counterstained with DAPI (0.025 μg/ml) for 15 min. Cells were acquired with Amnis Imagestream Mark II imaging flow cytometer (EMD Millipore) and analyzed with IDEAS v6.2 software. Polarity was determined using delta centroid and modulation morphology features for Cdc42, while β‐catenin nuclear translocation was determined by similarity with DAPI (nuclear translocation feature), similar to what we have previously published (Kwarteng et al., 2018). See Table 1 for additional details on antibodies.

### Colony Assays

BM single-cell suspensions were prepared in IMDM (Life Technologies) containing 10% Premium FBS (Wisent Bio Products, Saint-Bruno, QC, Canada). Using a syringe fitted with a blunt-ended needle, cells were plated in duplicates into 35-mm non-adherent Petri dishes at a density of 2 × 10^4^ cells/dish in methylcellulose medium optimized for multilineage erythro-myeloid growth (MethoCult™ GF M3434; Stem Cell Technologies). Cultures were incubated at 37 C in 5% CO_2_ for 10–14 days and hematopoietic colonies were counted and identified based on morphology under an inverted microscope. Cells were harvested by pooling duplicates for flow cytometry analysis.

### Statistical Analysis

Each graph represents at least three independent experiments, unless otherwise indicated. Two-tailed Student’s t test was used to determine statistical significance unless otherwise noted. A *p* value of 0.05 or less was considered significant.

## Results

### Vangl2 Regulates BM Multipotent Progenitor Cell Numbers and Spleen Size in Old Mice

**FIGURE 1:**
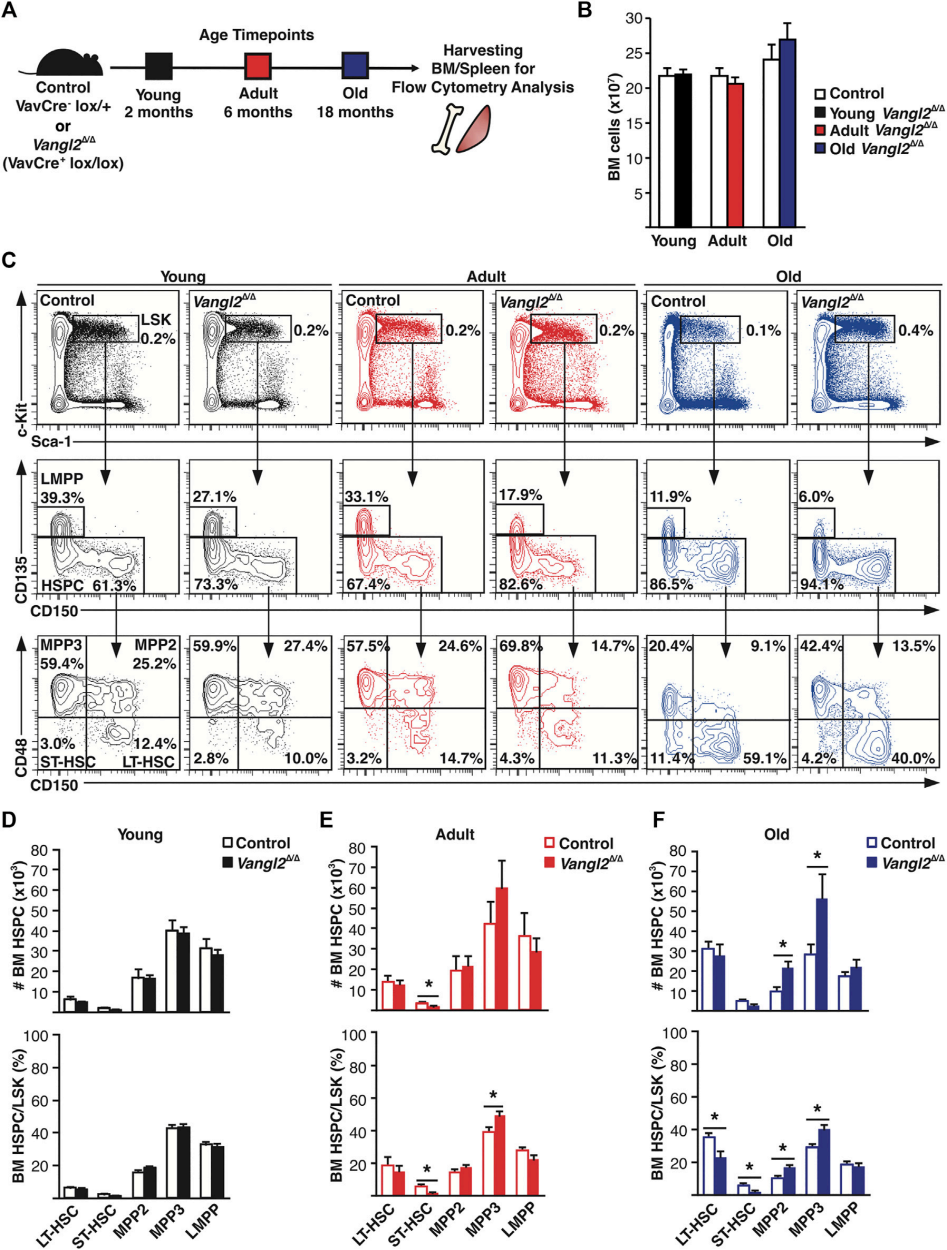
Vangl2 loss in hematopoietic cells increases myeloid-biased multipotent progenitor cell numbers in old mice. **(A)** Experimental design for flow cytometry analysis of young (2-month-old, in black), adult (6-month-old, in red) and old (18-month-old, in blue) VavCre^+^Vangl2^lox/lox^ mice in which Vangl2 is ablated in all hematopoietic lineages (*Vangl2* ^Δ/Δ^) and littermate controls. **(B)** Histogram represents absolute numbers of BM cells per leg (tibia + femur) for young, adult and old mice (mean +SEM). **(C)** Representative flow cytometry data are shown for young, adult and old mice. Populations were defined as follows: Lin^−^Sca1^+^c-Kit^+^ (LSK), CD135^+^CD48^+^CD150^−^ (LMPP), CD135^−^CD48^+^CD150^−^ (MPP3), CD135^−^CD48^+^CD150^+^ (MPP2), CD135^−^CD48^−^CD150^−^ (ST-HSC) and CD135^−^CD48^−^CD150^+^ (LT-HSC). Numbers within panels represent cell percentage for the panel shown. **(D-F)** Quantitative results of HSCs and progenitor cells flow cytometry analysis are represented in **(D)** for young, **(E)** adult, and **(F)** old mice. Histograms represent absolute numbers of BM cells in top panels and stem/progenitor cell percentage within the LSK pool in bottom panels (mean +SEM). See also Supplementary Figure S1. Young (n = 17 control, 18 *Vangl2* ^Δ/Δ^), adult (n = 10 control, 11 *Vangl2* ^Δ/Δ^), old (n = 14 control, 14 *Vangl2* ^Δ/Δ^), *p ≤ 0.05.

To investigate the functional role of Vangl2 in homeostatic conditions, we first analyzed BM hematopoiesis using a mouse model in which the polarity gene *Vangl2* is inactivated in all hematopoietic lineages using the *Vav-iCre-LoxP* system (*Vangl2* ^Δ/Δ^ mice) (de Boer et al., 2003). We analyzed mice from both sexes and different age groups by flow cytometry (Figure 1A). There were no major differences in total BM cellularity between *Vangl2* ^Δ/Δ^ mice and controls in any age group, although it tended toward an increase in older (18-month-old) mice (Figure 1B). However, old *Vangl2* ^Δ/Δ^ mice presented a significant increase in total Lin^−^Sca-1^+^cKit^+^ (LSK) cell numbers driven by the expansion of myeloid-biased multipotent progenitor cell populations (MPP2 and MPP3) compared to their littermate controls (Figures 1C,F). Most of these changes were not seen in young (2-month-old) and adult (6-month-old) *Vangl2* ^Δ/Δ^ groups, although an increase in MPP3 frequency was already detectable in adult mice (Figures 1D,E). Similar tendencies were observed in both sexes (Supplementary Figure S1A,B,C); there was a significant decrease in the number of CD150^+^ CD48^−^ LT-HSCs in *Vangl2* ^Δ/Δ^ males compared to *Vangl2* ^Δ/Δ^ females, but the difference was not significant when compared to their sex-matched controls (Supplementary Figure S1C). Taken together, these results indicate that Vangl2 is dispensable for HSC emergence and their steady-state maintenance. However, the expansion of myeloid-biased MPPs in older mice suggest that Vangl2 may play a role in hematopoiesis, including age-dependent alterations in BM progenitor cell pool.

**FIGURE 2:**
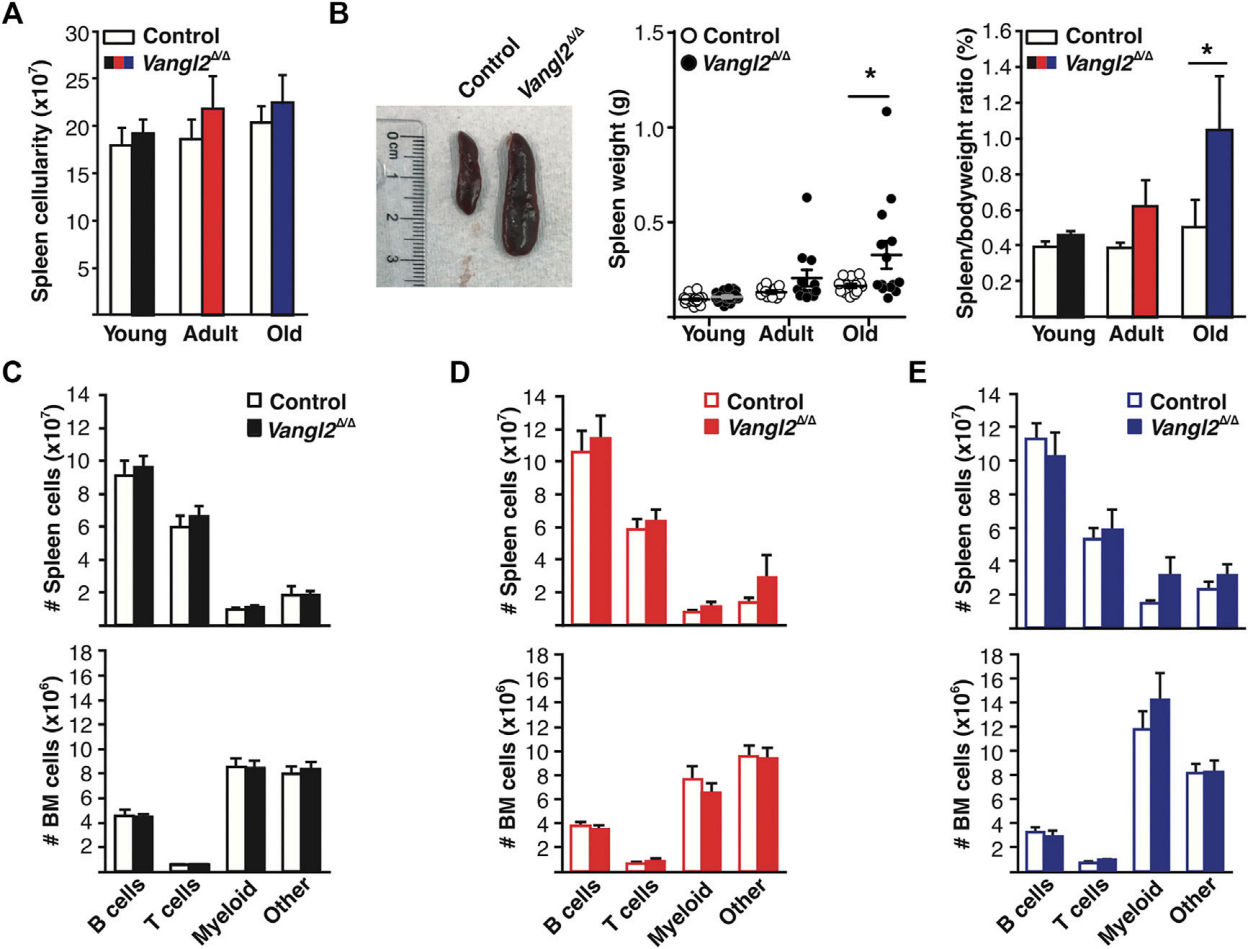
Vangl2 is dispensable for steady-state lympho-myelopoiesis, but its absence promotes splenomegaly in old mice. **(A)** Spleen cellularity for young (2-month-old), adult (6-month-old) and old (18-month-old) mice (mean +SEM). **(B)** Photograph of an enlarged *Vangl2* ^Δ/Δ^ spleen (left). Spleen weight for individual mice (middle; horizontal lines represent mean and error bars, SEM) and average spleen index as determined by the spleen weight (g)/bodyweight (g) ratio (right; mean +SEM). **(C-E)** Histograms represent absolute spleen (top panels) and BM (bottom panels) B lymphocyte (CD19^+^), T lymphocyte (CD3ε^+^), and myeloid (CD11b^hi^) cell numbers for **(C)** young mice, **(D)** adult mice, and **(E)** old mice (mean +SEM). CD19^−^ CD3ε^−^ CD11b^−/lo^ cells are represented as “Other”. See also Supplementary Figure S1,S2. Young (n = 17 control, 19 *Vangl2* ^Δ/Δ^), adult (n = 10 control, 11 *Vangl2* ^Δ/Δ^), old (n = 14 control, 14 *Vangl2* ^Δ/Δ^), *p ≤ 0.05.

Vangl2 is highly expressed by HSCs and progenitor cells compared to mature blood cells (Choi et al., 2019). Hence, to evaluate the role of Vangl2 in differentiation, we next analyzed mature lymphoid and myeloid cells in the BM and spleen (Supplementary Figure S2A,B). We found no differences in total spleen cellularity between control and *Vangl2* ^Δ/Δ^ mice (Figure 2A). However, we noted a significant increase in spleen size and weight in a subset of old *Vangl2* ^Δ/Δ^ mice (Figure 2B). This was not driven by an overall increase in body size, as similar differences were also seen using spleen index (spleen/bodyweight ratio; Figure 2B), and it occurred more frequently in females (5/7 at 18 months and 3/6 at 6 months) than in males (1/6 and 0/6, respectively).

Although the proportion of myeloid cells increased with age, as expected, there was no significant difference in the number of myeloid cells in the BM or spleen between *Vangl2* ^Δ/Δ^ mice and age-matched controls (Figures 2C–E), irrespective of sex (Supplementary Figure S1D-F). However, there was a tendency toward an increased accumulation of myeloid cells in the spleen of old *Vangl2* ^Δ/Δ^ mice (Figure 2E) that appeared mostly restricted to females (Supplementary Figure S1F), in line with the stronger MPP expansion in the BM of *Vangl2* ^Δ/Δ^ females (Supplementary Figure S1C) and the occurrence of splenomegaly.

### *Vangl2*-Deficient Progenitor Cells Show Impaired Expansion in Culture

**FIGURE 3:**
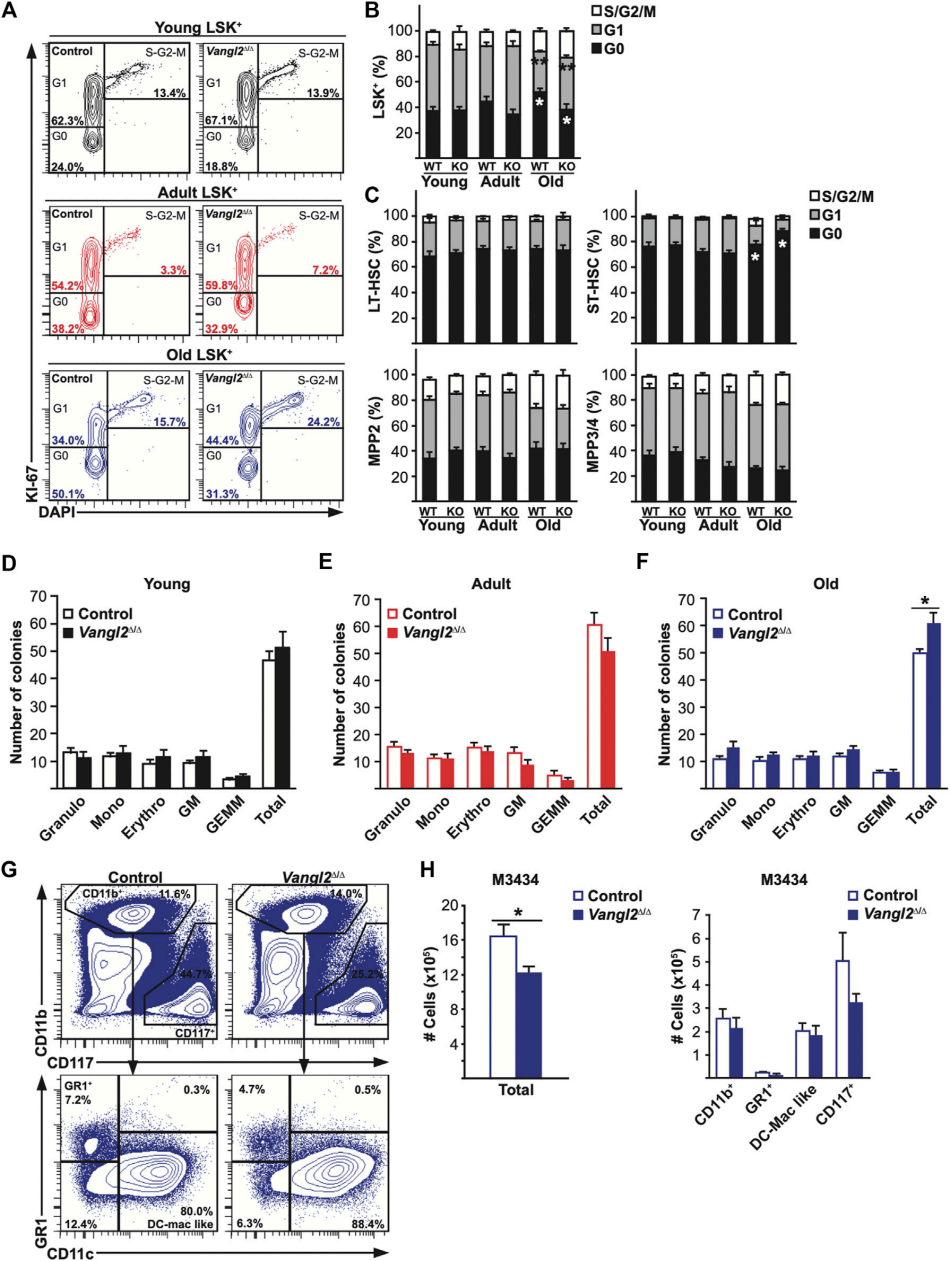
Vangl2 loss impairs old myeloid progenitor cell expansion *in vitro*. **(A)** Representative flow cytometry data are shown for LSK^+^ cells from young (2-month-old), adult (6-month-old) and old (18-month-old) mice. Numbers within panels represent G0, G1 and S-G2-M cell percentage for the panel shown. **(B and C)** Quantitative results of flow cytometry cell cycle analysis are represented for young, adult and old **(B)** LSK + cells and **(C)** LT-HSC, ST-HSC, MPP2 and MPP3/4 cells (mean +SEM). Young (n = 10 control, 12 *Vangl2* ^Δ/Δ^), adult (n = 8 control, 10 *Vangl2* ^Δ/Δ^), old (n = 8 control, 10 *Vangl2* ^Δ/Δ^). **(D-F)** Number of colonies counted after 10–14 days *in vitro* are represented for **(D)** young (n = 8), **(E)** adult (n = 5) and **(F)** old (n = 5) BM cells. Granulo: granulocyte, Mono: monocyte, Erythro: erythroid, GM: granulocyte/macrophage, GEMM: granulocyte/erythroid/macrophage/megakaryocyte. **(G)** Representative flow cytometry data of harvested colony-forming units **(H)** Histograms represent cell numbers recovered per pooled duplicate Petri dishes seeded with BM cells from old mice (mean +SEM). *p ≤ 0.05, **p ≤ 0.005.

The increase in BM multipotent progenitors in older *Vangl2* ^Δ/Δ^ mice led us to investigate the proliferative activity of *Vangl2* ^Δ/Δ^ stem/progenitor cells. To evaluate cell cycle, we performed a Ki-67 and DAPI intracellular staining and analyzed different cell cycle phases by flow cytometry (Szade et al., 2016). As expected, we found no differences between controls and *Vangl2* ^Δ/Δ^ mice in young and adult groups (Figure 3A, top and middle panel). However, we did find differences in the old group in which *Vangl2* ^Δ/Δ^ mice had fewer quiescent LSKs and more LSKs in G_1_ phase (Figure 3B). However, no differences were seen within most sub-populations, except for an increase in the proportion of *Vangl2* ^Δ/Δ^ ST-HSCs in G_0_ phase (Figure 3C). This apparent discrepancy can be explained by the relative increase in the frequency of MPPs (that are actively cycling) concomitant with the relative decrease in HSCs (that are generally more quiescent) within the *Vangl2* ^Δ/Δ^ LSK population as compared to control LSKs (Figures 1C,F).

To further determine whether their differentiation *in vitro* was affected, we evaluated BM myeloid colony-forming units after 10–14 days of culture. As expected, we did not see any differences in young and adult mice (Figures 3D,E), but there was a slight increase in the total number of colonies from old *Vangl2* ^Δ/Δ^ BM (Figure 3F). Flow cytometry analysis revealed that although more colonies were generated by old *Vangl2* ^Δ/Δ^ mice, they consisted on average of fewer cells (Figures 3G,H). This was consistent with a decreased frequency of CD117^+^ cells that are normally found in colonies with high proliferative potential (Figures 3G,H). These results suggest Vangl2-deficient old progenitor cells have a decreased proliferative capacity *in vitro*, despite their being no significant changes in their overall cell cycle status.

### Vangl2 Is Required for Post-transplant Peripheral Blood and BM Reconstitution in Adult Donors

**FIGURE 4:**
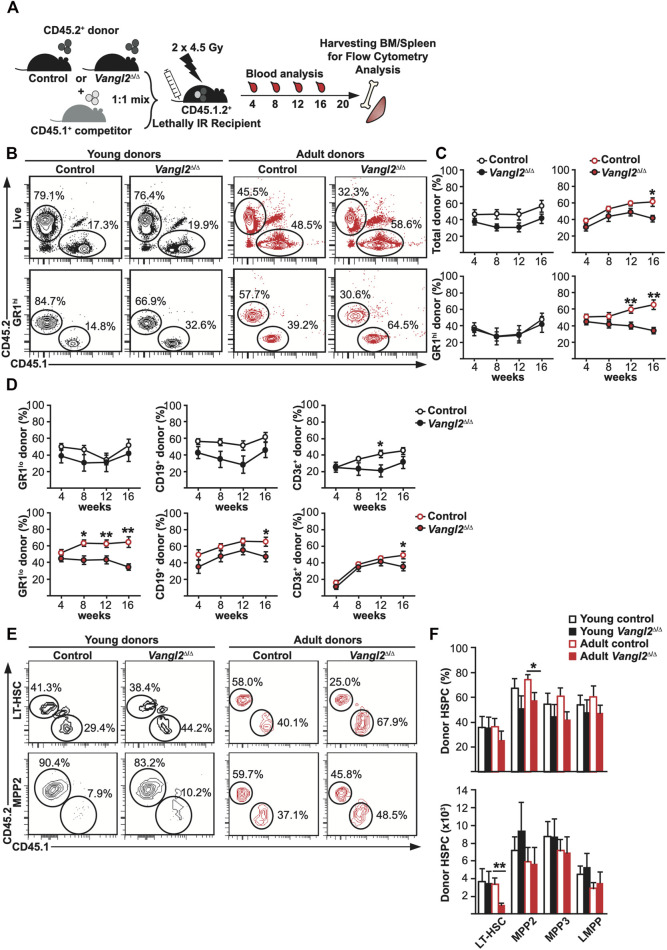
Vangl2 is required for hematopoietic reconstitution in adult donors. **(A)** Experimental design for primary transplants **(B)** Representative flow cytometry data showing the percentage of donor-derived total and GR1^hi^ cells in recipient peripheral blood post-transplant for young (2-month-old) and adult (6-month-old) donors at 16 or 12 weeks post-transplant, respectively. **(C)** Graphs represent pooled results of donor-derived total and GR1^hi^ cells in recipient peripheral blood for young and adult donors (mean +SEM). **(D)** Graphs represent pooled results of donor-derived GR1^lo^ cells, CD19^+^ B cells and CD3ε^+^ T cells in recipient peripheral blood for young and adult donors (mean +SEM). **(E)** Representative flow cytometry data showing the percentage of CD45.2^+^ donor-derived or CD45.1^+^ competitor LT-HSCs (top panels) and MPP2s (bottom panels) in recipient BM 20 weeks post-transplant for young and adult donors. **(F)** Histograms represent mean of young or adult donor-derived HSPCs chimerism (top) or absolute cell numbers (bottom) in BM 20 weeks post-transplant (mean +SEM). See also Supplementary Figure S2,S3,S4. Data for transplants with young female donors are pooled from three independent experiments (n = 14–16 control, 13–15 *Vangl2* ^Δ/Δ^) and for adult female donors, from two independent experiments (n = 10 control, 11 *Vangl2* ^Δ/Δ^). *p ≤ 0.05, **p ≤ 0.005.

Considering differences seen in homeostatic conditions in *Vangl2* ^Δ/Δ^ mice, we next evaluated HSC functionality under hematopoietic pressure. The gold-standard assay for HSC function is to evaluate their ability to reconstitute a lethally irradiated host so we conducted competitive bone marrow transplant assays as described (Kwarteng and Heinonen, 2016; Hétu-Arbour et al., 2021a) and followed post-transplant recovery by analyzing peripheral blood reconstitution for 16 weeks and BM recovery at 20–22 weeks by flow cytometry (Figure 4A). To evaluate the possible age-related role of Vangl2, we performed BM transplants with young (2-month-old) and adult (6-month-old) donors. We found no differences in recovery in primary recipient mice transplanted with young *Vangl2* ^Δ/Δ^ BM cells. However, we observed a significant decrease in overall peripheral blood chimerism after 16 weeks in mice transplanted with adult *Vangl2* ^Δ/Δ^ BM cells compared to their controls (Figures 4B,C; Supplementary Figure S2C). Furthermore, mice transplanted with adult *Vangl2* ^Δ/Δ^ BM cells showed a significant decrease in donor-derived GR1^hi^ granulocytes (12–16 weeks post-transplant), GR1^lo^ monocytes (8–16 weeks post-transplant), CD19^+^ B cells (16 weeks post-transplant) and CD3ε^+^ T cells (16 weeks post-transplant) in peripheral blood (Figures 4C,D). We next evaluated BM HSC and progenitor cell recovery 20 weeks post-transplant. As expected from peripheral blood analysis, no differences were seen in mice transplanted with young *Vangl2* ^Δ/Δ^ vs. control BM cells. However, mice transplanted with adult *Vangl2* ^Δ/Δ^ BM cells had a significant decrease in donor-derived BM LT-HSCs as compared to controls (Figures 4E,F). No differences were observed in mature cell numbers in BM or spleen in primary transplant recipients at 20 weeks (Supplementary Figure S3A,B).

### *Vangl2* ^Δ/Δ^ BM Cells Fail in Serial Transplants, Leading to Hematopoietic Failure

**FIGURE 5:**
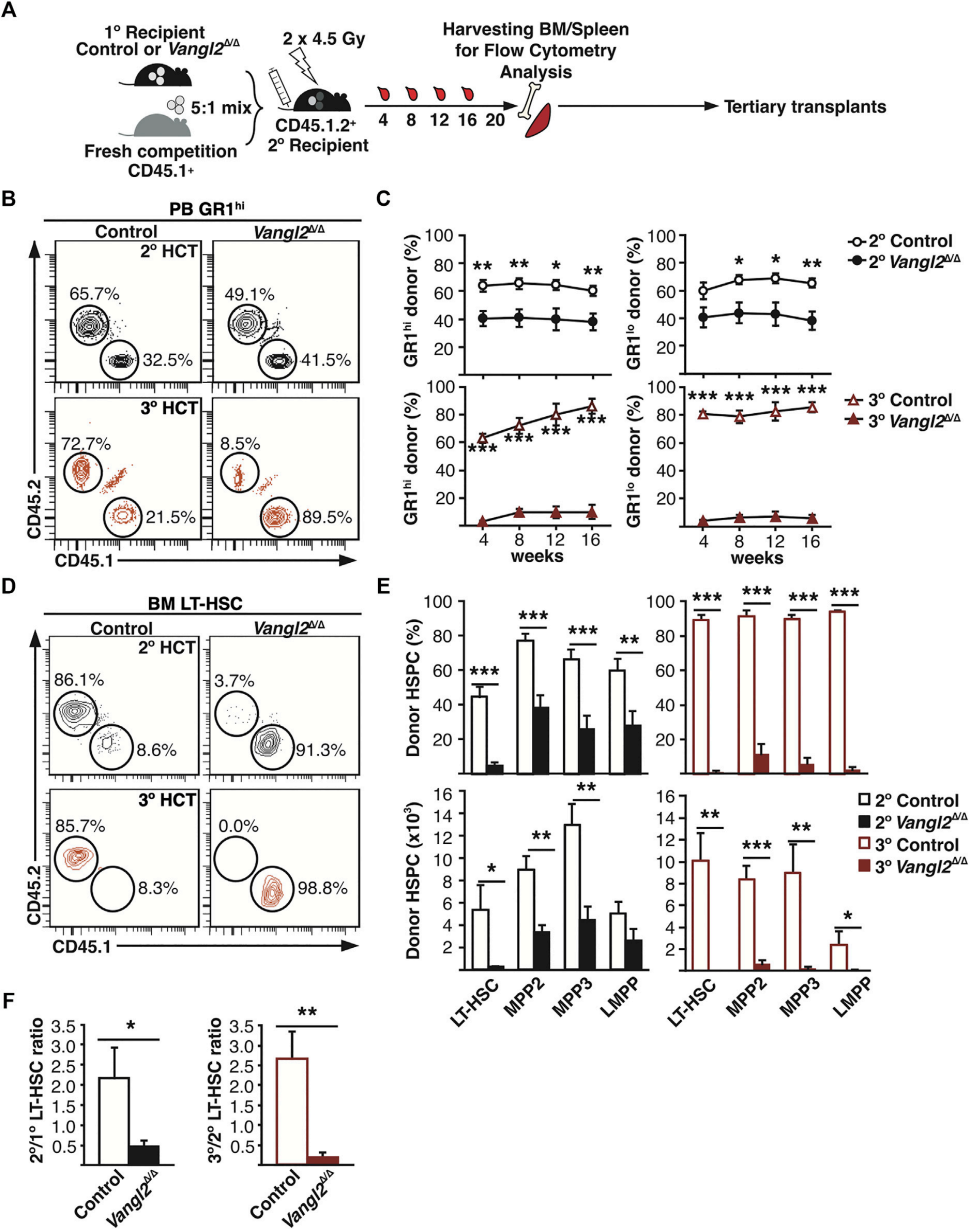
Vangl2 deficiency severely impairs hematopoietic recovery in serial transplants, leading to BM failure. **(A)** Experimental design for serial transplants **(B)** Representative flow cytometry data showing the percentage of donor-derived GR1^hi^ cells in secondary and tertiary recipient peripheral blood 16 weeks post-transplant. **(C)** Graphs represent pooled results of donor-derived GR1^hi^ granulocytes and GR1^lo^ monocytes in secondary and tertiary recipient peripheral blood (mean +SEM). **(D)** Representative flow cytometry data showing the percentage of CD45.2^+^ donor-derived or CD45.1^+^ competitor LT-HSCs in BM of secondary (top panels) and tertiary recipients (bottom panels) 20 weeks post-transplant. **(E)** Histograms represent donor chimerism or absolute donor-derived cell numbers in secondary/tertiary recipient BM 20 weeks post-transplant (mean +SEM). **(F)** Donor-derived LT-HSC ratio (post-/pre-transplant) 20 weeks post-transplant. See also Supplementary Figure S2,S3,S4. *p ≤ 0.05, **p ≤ 0.005, ***p ≤ 0.0005. Secondary transplants represent two independent transplant groups (n = 9 control, 10 *Vangl2* ^Δ/Δ^), while tertiary transplants represent one single group (n = 4 control, six *Vangl2* ^Δ/Δ^).

To evaluate *Vangl2* ^Δ/Δ^ HSC long-term self-renewal capacity, we then performed serial BM transplantation assays using primary recipients of young BM as donors (Figure 5A). Secondary transplants from primary *Vangl2* ^Δ/Δ^ donors showed a significant decrease in myeloid (GR1^hi^ and GR1^lo^) long-term reconstitution in peripheral blood as compared to controls (Figures 5B,C in black). This was most likely due to a functional impairment in the *Vangl2* ^Δ/Δ^ stem/progenitor cell compartment as we did not detect any differences in the frequency or number of phenotypic donor HSCs in the primary recipient BM (Figures 4E,F in black). We also observed a significant decrease in *Vangl2* ^Δ/Δ^ donor-derived LT-HSC, MPP2, MPP3 and LMPP numbers in secondary recipient BM (Figures 5E,F). *Vangl2* ^Δ/Δ^ donor-derived mature B and T cell numbers were not decreased as compared to controls in peripheral blood, spleen or BM of secondary recipients (Supplementary Figure S3C-F), most likely due to their long half-life. This was also reflected in total donor chimerism in peripheral blood (Supplementary Figure S3C,D), which is consistent with lymphocytes representing up to 80% leukocytes in murine peripheral blood (O'Connell et al., 2015; Stahl et al., 2018). In contrast, short-lived *Vangl2* ^Δ/Δ^ donor-derived GR1^hi^ granulocytes dependent on constant replacement showed a significant decrease in spleen as well as in BM (Supplementary Figure S3E,F) that on average was at least as strong as that observed in peripheral blood at slightly earlier time points (approximately two-fold). Donor chimerism in BM MPPs at 20 weeks was very similar to what was seen in the periphery for myeloid cells 16 weeks post-transplant, and this applied to both *Vangl2* ^Δ/Δ^ and control donors (Figures 5C,E); however, the proportion of donor-derived HSCs was significantly lower (Figures 5D,E), particularly in the case of *Vangl2* ^Δ/Δ^ donors. The direct contribution of LT-HSCs to steady state hematopoiesis remains under debate (Sun et al., 2014; Chapple et al., 2018), but progenitor cells with little to no transplantation capacity have been shown to support local granulocyte production for up to a year (Sun et al., 2014), which would explain our result.

To further corroborate our interpretation, tertiary transplants with *Vangl2* ^Δ/Δ^ BM cells displayed a nearly complete failure of hematopoietic reconstitution as little to no *Vangl2* ^Δ/Δ^ donor-derived cells were observed from 4–16 weeks in myeloid or lymphoid lineages (Supplementary Figure S5B,C and Supplementary Figure S3C,D in red). The low frequency of donor-derived LT-HSCs in the graft certainly contributed to the impaired reconstitution in tertiary transplants, but the proportion of donor-derived cells was even lower than what would be mathematically expected (approx. 10% CD45.2^+^ LT-HSCs in the secondary recipients used as BM donors), further confirming the decline not only in phenotypic but also in functional HSCs in the *Vangl2* ^Δ/Δ^ donor cell pool. In comparison, control donor-derived cells represented up to 80% total peripheral blood, an increase from what was seen in the BM of secondary recipients (approx. 40% CD45.2^+^ LT-HSCs). Similar results were seen in BM and spleen, where *Vangl2* ^Δ/Δ^ cells were essentially absent except for the myeloid lineage (Supplementary Figure S3E,F). This corresponded to the presence of a detectable *Vangl2* ^Δ/Δ^ donor-derived population only within MPP2 and MPP3 compartments in the BM (Figures 5E,F), suggesting that these cells could still maintain traces of myelopoiesis in tertiary recipients. In an attempt to account for the differences in donor cell input, especially in tertiary transplants, we compared donor LT-HSC numbers from the graft to those recovered 20 weeks post-transplant (number of cells in recipient BM/number of cells injected). We found no expansion of *Vangl2* ^Δ/Δ^ LT-HSCs in secondary or tertiary transplants, resulting in net loss of cells and the absence of *Vangl2* ^Δ/Δ^ LT-HSCs in tertiary transplants (Figure 5F). In comparison, control LT-HSCs retained their capacity to expand (approximately 2.5-fold in both cases). Similar results were obtained with male mice with the exception of slightly improved *Vangl2* ^Δ/Δ^ GR1^hi^ granulocyte reconstitution in peripheral blood following primary transplants; however, donor contribution was significantly decreased in all lineages following secondary transplants (Supplementary Figure S4A,B).

### *Vangl2*-Deficiency Increases p16^INK4a^ Expression in LT-HSCs and Promotes β-catenin Stabilization

**FIGURE 6:**
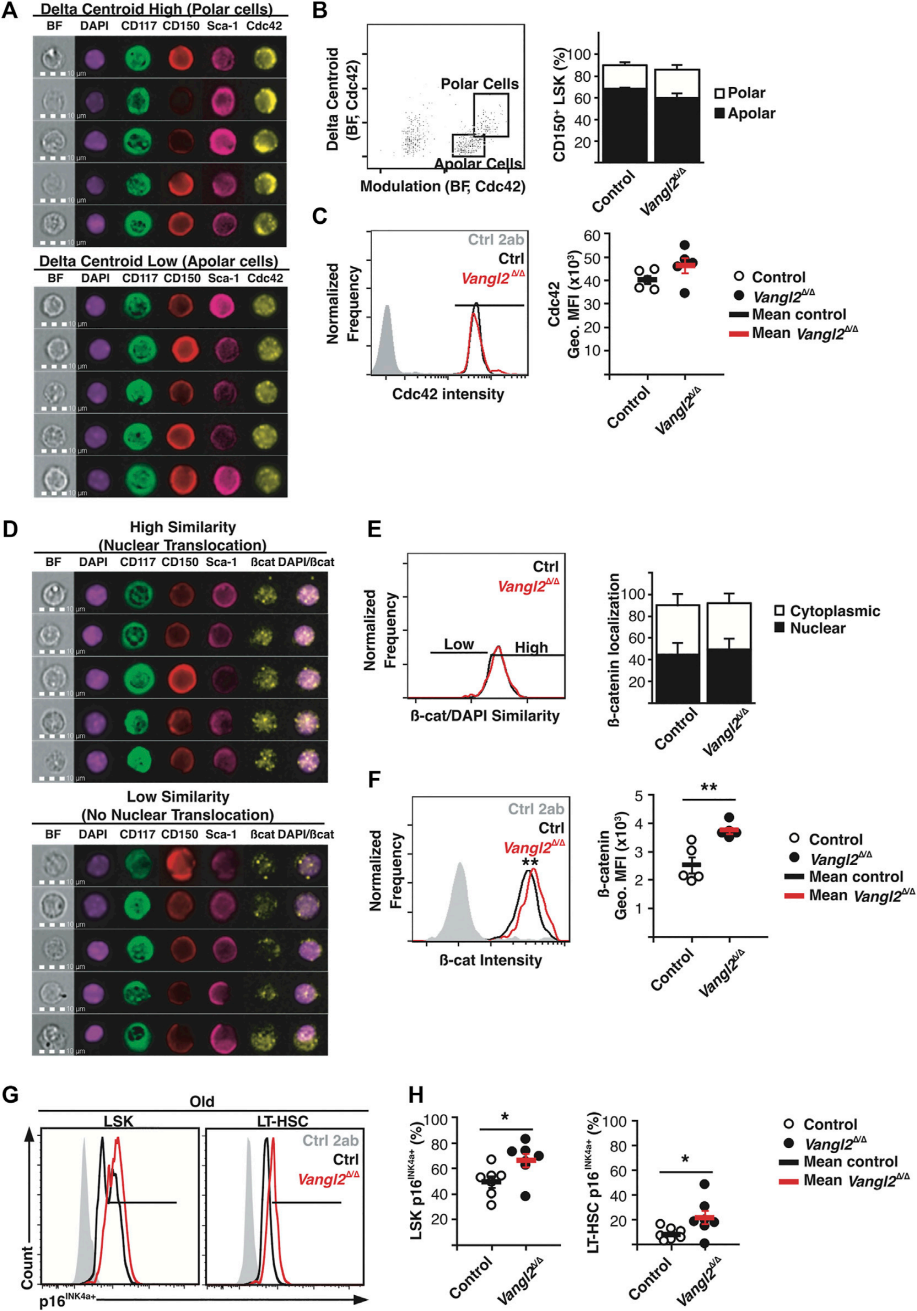
Vangl2 loss increases β-catenin activation and p16INK4a expression in old stem/progenitor cells. **(A)** Representative images of polar and apolar CD150^+^ LSK^+^ cells **(B)** Gating strategy based on Cdc42 delta centroid and morphology modulation features. Histogram represents the frequency of polar and apolar CD150^+^ LSK^+^ cells from old mice (mean +SEM). **(C)** Representative overlay of Cdc42 staining intensity (left) and quantification of Cdc42 geometric mean of fluorescence intensity (Geo. MFI). **(D)** Representative images of β-catenin (β-cat) nuclear translocation based on β-catenin/DAPI similarity analysis. **(E)** Gating strategy based on β-cat/DAPI similarity dilate analysis. Histogram represents cytoplasmic and nuclear β-catenin localization in old CD150^+^ LSK^+^ cells (mean +SEM). **(F)** Representative overlay of β-catenin staining intensity (left) and quantification of β-catenin geometric mean of fluorescence intensity (Geo. MFI) in old CD150^+^ LSK^+^ cells. **(G)** Representative overlay of p16^INK4a^ expression in old LSK^+^ cells and LT-HSCs **(H)** Graphs represent the frequencies of p16^+^ cells within LSK^+^ and LT-HSC compartments (mean +SEM). See also Supplementary Figure S5. *p ≤ 0.05 (n = five to six, three independent experiments).

Since Wnt/PCP and Vangl2 signalling is known to control polarity in other cell types and because establishment of polarity is important for HSC self-renewal (Florian et al., 2018), we next investigated if Vangl2 loss impaired HSC polarity. To do so, we analyzed the cellular distribution of Cdc42, a small GTPase and polarity marker in HSCs, with imaging flow cytometry (Figure 6A) (Florian et al., 2012). We found no significant differences in polar and apolar CD150^+^ LSKs or Cdc42 intensity in old *Vangl2* ^Δ/Δ^ mice (Figures 6B,C). Similarly, no differences were found in young and adult mice, suggesting Vangl2 does not play a role in Cdc42 activation or localisation (Supplementary Figure S5A,B). However, as previously shown by others (Florian et al., 2012), cells from old mice were more frequently apolar.

Non-canonical Wnt signalling can inhibit the canonical Wnt/β-catenin pathway (Nemeth et al., 2007), so we next evaluated β-catenin stabilization and localization (Figure 6D). Nuclear *versus* cytoplasmic β-catenin ratios were not altered in old *Vangl2* ^Δ/Δ^ mice (Figure 6E). Similar results were found with young and adult mice (Supplementary Figure S5C,D). However, we observed a significant increase in β-catenin mean fluorescent intensity within old *Vangl2* ^Δ/Δ^ CD150 + LSKs as compared to controls, suggesting a potentially higher β-catenin activity in old *Vangl2* ^Δ/Δ^ cells (Figure 6F, Supplementary Figure S5E).

Prolonged Wnt/β-catenin signalling is thought to induce senescence in other cell types (Gu et al., 2014), and it has been linked to HSC exhaustion and hematopoietic failure (Scheller et al., 2006). Cyclin-dependent kinase inhibitor and cell cycle regulator p16^INK4a^ is often used as a marker of senescence, and although its endogenous expression is not uniformly associated with normal HSC aging (Janzen et al., 2006; Attema et al., 2009), enforced p16^INK4a^ upregulation is associated with loss of HSC self-renewal (Oguro et al., 2006). There was a significant increase in p16^INK4a^ expression among LSKs as well as LT-HSCs in old *Vangl2* ^Δ/Δ^ BM, suggesting p16^INK4a^ activation within the BM progenitor cell pool in old *Vangl2* ^Δ/Δ^ mice (Figures 6G,H), which may contribute to their impaired recovery post-transplant.

## Discussion

The importance of Wnt signalling in HSC self-renewal and hematopoiesis has been widely studied and debated, and while an equilibrium between the various intracellular pathways promotes the most optimal conditions, how this equilibrium is actually maintained remains still largely undefined. Although inhibition of Wnt binding to its receptors impairs HSC self-renewal and differentiation (Fleming et al., 2008; Renström et al., 2009; Schaniel et al., 2011), Wnt glycosylation and secretion is reportedly dispensable for adult hematopoiesis (Kabiri et al., 2015). Given the large number and potential promiscuity of Wnt ligands combined with the variety of hematopoietic and non-hematopoietic BM cells producing them, we chose to focus our study on the Wnt/PCP coreceptor Vangl2. Wnt/PCP components have been recently shown to be important in HSC maintenance and self-renewal (Sugimura et al., 2012; Abidin et al., 2015; Lhoumeau et al., 2016). Despite being highly expressed by HSCs and progenitor cells (Choi et al., 2019), Vangl2 is best known for its crucial role in neural tube development and cell polarity (Kibar et al., 2001; Murdoch et al., 2001; Montcouquiol et al., 2003). Our results demonstrate an essential role for Vangl2 in HSC expansion and hematopoietic recovery post-transplant and suggest it could play a role in regulating age-associated hematopoietic alterations.

Hematopoietic aging is associated with the accumulation of cells expressing HSC markers but having more limited self-renewal capacity on an individual basis and presenting with a bias toward myeloid differentiation (Beerman et al., 2010; Dykstra et al., 2011; de Haan and Lazare, 2018). While Vangl2 was not required for normal hematopoiesis in young mice, aging 18-month-old *Vangl2* ^Δ/Δ^ mouse BM contained more myeloid-biased multipotent progenitor cells, and a similar trend could be observed in 6-month-old adults as well, suggesting a gradual effect of Vangl2 deletion in hematopoiesis that increases with age. This was not associated with an overall loss of HSC or progenitor cell quiescence, in contrast to what was reported for *Fzd8* ^−/−^ or *Celsr2/Fmi* ^−/-^ HSCs (Sugimura et al., 2012), as we observed no significant increase in Ki-67^+^ cycling cells in any age group. To the contrary, there was a slight but significant increase in the proportion of quiescent *Vangl2* ^Δ/Δ^ ST-HSCs, and *Vangl2* ^Δ/Δ^ HSCs and MPPs expressed increased levels of the cell cycle inhibitor p16^INK4a^. Although p16^INK4a^ may not play a major role in normal HSC aging, its enhanced expression impairs HSC self-renewal (Oguro et al., 2006; Attema et al., 2009). Inversely, the absence of p16^INK4a^ specifically enhances repopulation ability of BM cells from aged donors (Janzen et al., 2006). The absence of Vangl2 might thus exacerbate the effects of HSC aging by upregulating p16^INK4a^. Indeed, although there was a slight increase in the frequency of myeloid colony-forming cells in *Vangl2* ^Δ/Δ^ BM, in line with the accrued number of myeloid-biased MPPs, these colonies consisted of fewer cells, indicative of an attenuated proliferative potential. Moreover, while young *Vangl2* ^Δ/Δ^ BM remained functional in primary transplants, HSCs from 6-month-old *Vangl2* ^Δ/Δ^ donors showed decreased expansion and long-term myeloid reconstitution, demonstrating the impact of age on *Vangl2* ^Δ/Δ^ HSC function. Serial transplants with young BM donors further confirmed the decreased repopulating capacity of *Vangl2* ^Δ/Δ^ BM cells, suggesting that cell-intrinsic Vangl2 is required for HSC expansion and self-renewal post-transplant.

We have recently published that the deletion of Wnt4 in BM stem/progenitor cells also resulted in impaired hematopoietic recovery after transplant or sublethal irradiation and corresponded to an increased expression of p16^INK4a^ in CD150^+^ LSKs (Hétu-Arbour et al., 2021b). Wnt4 is a putative ligand for Vangl2 in other cell types (Messéant et al., 2017), and the similarities in the results from the two studies suggest that this could also be the case in hematopoietic cells. p16^INK4a^ upregulation in *Wnt4* ^Δ/Δ^ BM cells was further associated with increased JNK activity and cellular stress (Hétu-Arbour et al., 2021b). In addition to its role in vertebrate Wnt/PCP signaling, JNK is also induced in response to different types of cellular stress, and it can regulate p16^INK4a^ expression via JunB (Passegue and Wagner, 2000). Vangl2 has been shown to phosphorylate JNK (Gao et al., 2011; Brunt et al., 2021), but it remains to be determined if JNK activity is altered in *Vangl2* ^Δ/Δ^ BM cells.

HSC self-renewal is dependent not only on the frequency of cell division but also on the type of division that takes place. Young adult BM HSCs would preferentially undergo asymmetric self-renewal divisions, thus generating daughter cells ready for differentiation while at the same time maintaining the size of the stem cell pool (Wu et al., 2007; Florian and Geiger, 2010; Ting et al., 2012; Florian et al., 2013). Increased Wnt5a/Cdc42 signaling has been shown to promote functional aging via loss of HSC polarity (Florian et al., 2012; Florian et al., 2013; Florian et al., 2018), whereas Wnt/PCP receptors appear to favor HSC self-renewal (Sugimura et al., 2012; Abidin et al., 2015; Lhoumeau et al., 2016), similar to what we found here for Vangl2. It must be noted that the role of PCP signaling in establishing HSC polarity remains unclear, and we detected no striking differences in Cdc42 distribution between *Vangl2* ^Δ/Δ^ and control CD150^+^ LSKs *ex vivo*, although we observed an age-dependent decrease in Cdc42 polarization in both genotypes. Nevertheless, it remains possible that Vangl2-dependent differences do exist in a more restricted subset of HSCs, or that their detection requires contact with niche cells or extracellular matrix. It is also possible that the impact of Vangl2 on cell division symmetry is Cdc42-independent. Wnt/PCP signaling has been shown to promote symmetric self-renewal divisions in satellite cells by establishing an asymmetrical distribution of Vangl2 that was essential for stem cell expansion (Le Grand et al., 2009). Unfortunately, we have not been able to find a good commercially available antibody to investigate Vangl2 localization in HSCs.

HSC aging has also been linked to a decrease in canonical β-catenin-dependent Wnt signaling (Florian et al., 2013), even though the functional link between the two remains to be confirmed. Although β-catenin stabilization is necessary for BM recovery after myeloablation (Lento et al., 2014), it is generally considered dispensable for HSC maintenance at steady state (Cobas et al., 2004; Luis et al., 2011). Non-canonical Wnt signalling can inhibit the Wnt/β-catenin canonical pathway, either by preventing β-catenin stabilization or by interfering with its translocation to the nucleus (Nemeth et al., 2007). Nuclear vs. cytoplasmic distribution of β-catenin was not altered in *Vangl2* ^Δ/Δ^ CD150^+^ LSKs. However, the intensity of β-catenin staining in old *Vangl2* ^Δ/Δ^ cells was significantly increased, suggesting an increased activation of the canonical pathway. Although the vast majority of CD150^+^ LSKs are CD48^−^, they do not represent a homogenous population of functional HSCs, especially in older mice. Moreover, given that the increase in β-catenin was only seen in aged *Vangl2* ^Δ/Δ^ mice, it could also be the result of adaptative changes in the BM environment in the absence of Vangl2, rather than the lack of direct Vangl2-mediated negative signaling in HSCs. However, chronic Wnt/β-catenin signalling has been shown to lead to cell senescence in other cell types (Gu et al., 2014), and lead to loss of function and exhaustion in HSCs (Scheller et al., 2006; Luis et al., 2011). The increased stabilization of β-catenin could thus contribute to the loss of *Vangl2* ^Δ/Δ^ HSC self-renewal.

Taken together, we have shown in this study the functional importance of the key Wnt/PCP component Vangl2 in HSC self-renewal and post-transplant recovery. This loss of repopulating ability corresponds to increased β-catenin and p16^INK4a^ levels in Vangl2-deficient HSCs from aged mice, revealing new mechanisms by which Vangl2 regulates hematopoiesis and HSC function in an age-dependent manner.

## Data Availability

The raw data supporting the conclusions of this article will be made available by the authors, without undue reservation.
