# Supplementary material for: Vangl2 Promotes Hematopoietic Stem Cell Expansion
Source: Front Cell Dev Biol. 2022 Mar 24;10:760248. doi: 10.3389/fcell.2022.760248 (PMC8987925; doi:10.3389/fcell.2022.760248)
Supplement: Supplementary file 1 [file DataSheet1.PDF]

Supplementary Material

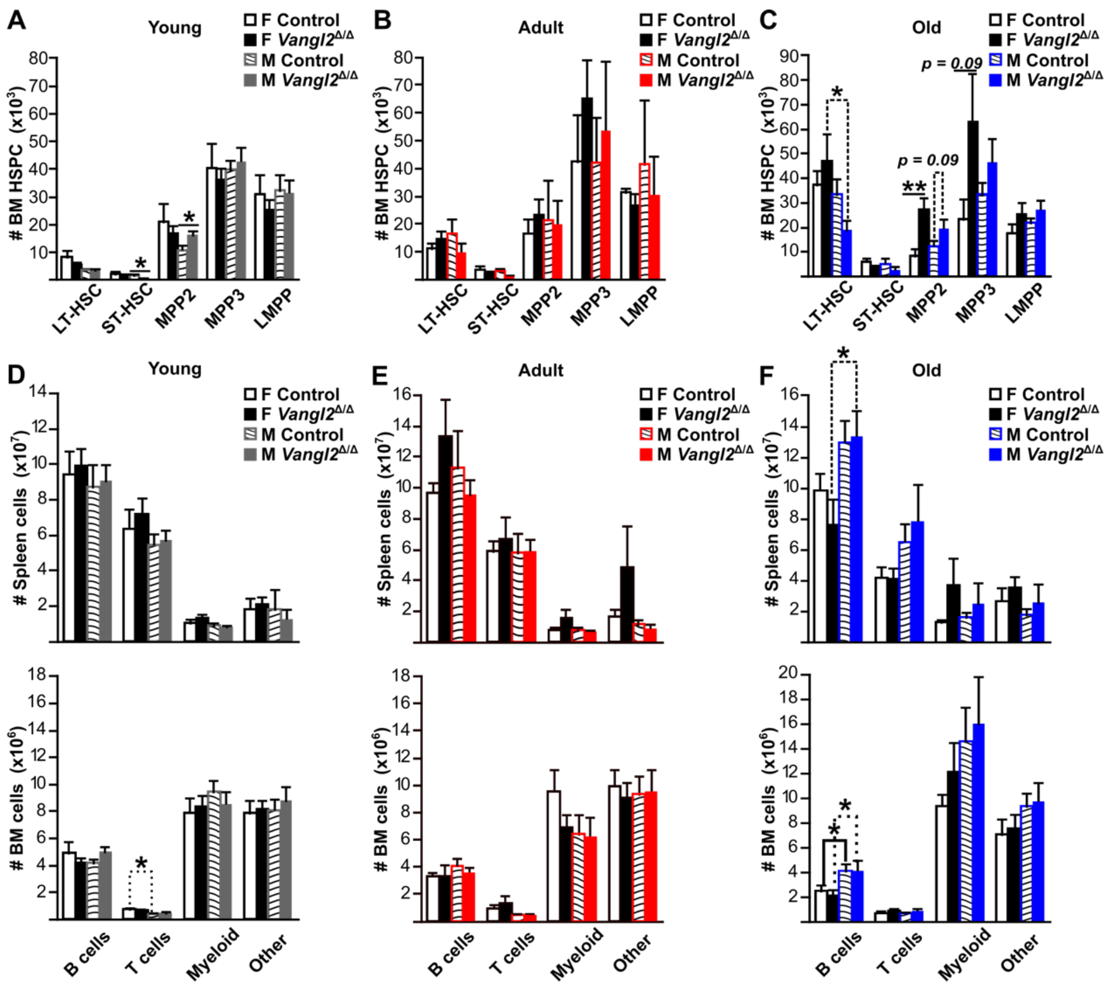

Supplementary Figure 1. Sex-related analysis of Vangl2 loss in HSCs, progenitor cells and mature BM/spleen cells in female and male mice, Related to Figures 1 and 2.

(A-C) Quantitative results of HSCs and progenitor cells flow cytometry analysis are represented in **(A)** for young, **(B)** adult, and **(C)** old females and males. Histograms represent absolute BM cell numbers (mean + SEM). **(D-F)** Histograms represent absolute spleen (top panels) and BM (bottom panels) B lymphocyte ($CD19^{+}$), T lymphocyte ($CD3\varepsilon^{+}$), and myeloid ($CD11b^{hi}$) cell numbers for **(D)** young mice, **(E)** adult mice, and **(F)** old mice (mean + SEM). $CD19^{-}CD3\varepsilon^{-}CD11b^{-/lo}$ cells are represented as "Other". Young females (n=10 control, 11-12 *Vangl2*$^{\Delta/\Delta}$), young males (n=7 control, 7 *Vangl2*$^{\Delta/\Delta}$), adult females (n=5 control, 6 *Vangl2*$^{\Delta/\Delta}$), adult males (n=5 control, 5 *Vangl2*$^{\Delta/\Delta}$), old females (n=7 control, 7 *Vangl2*$^{\Delta/\Delta}$), old males (n=6 control, 6 *Vangl2*$^{\Delta/\Delta}$). **p*≤0.05, ***p*≤0.005.

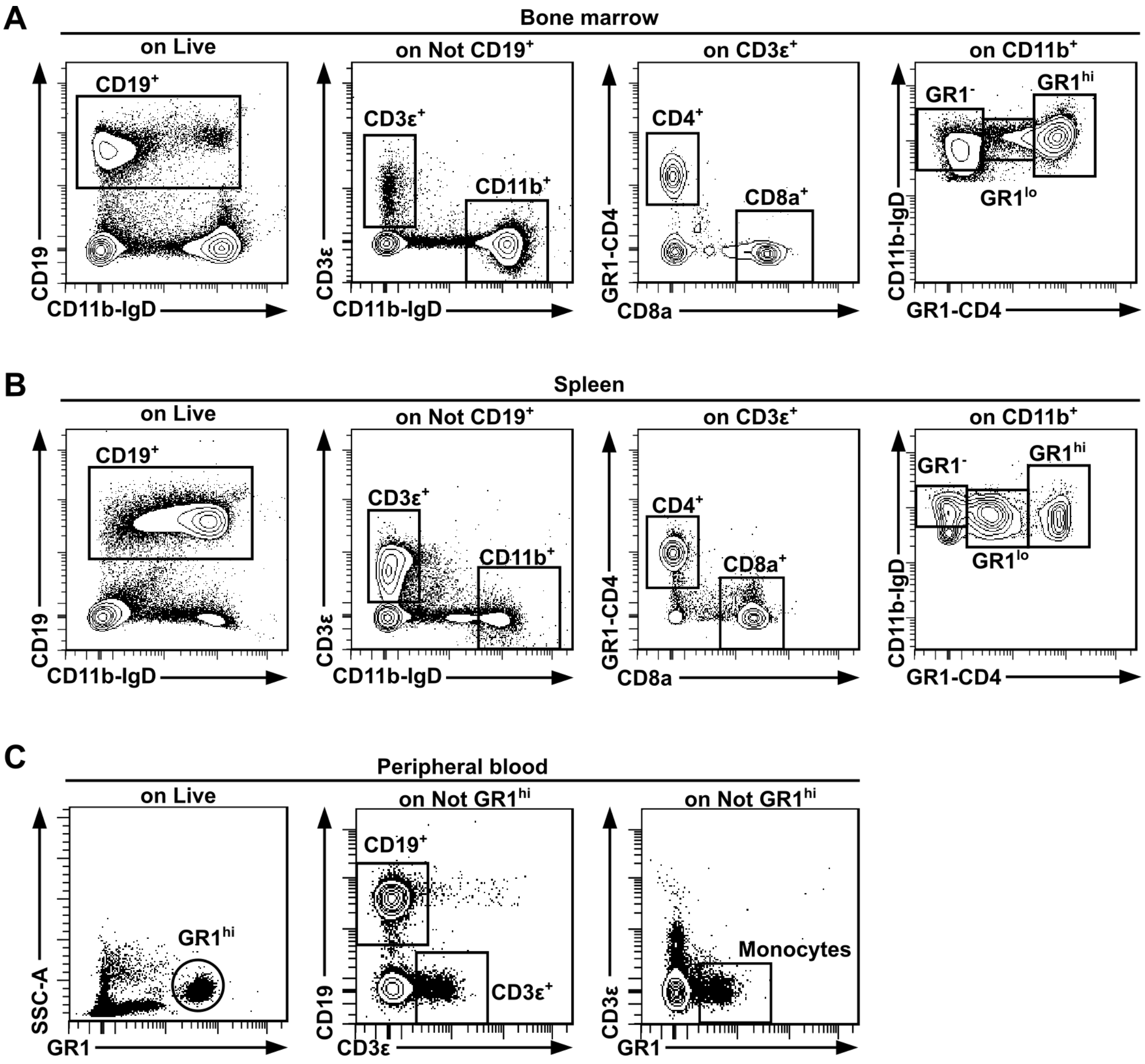

Supplementary Figure 2. Flow cytometry gating strategies, Related to Figures 2, 4 and 5.
(A and B) **(A)** BM and **(B)** spleen mature cells gating strategy. Within live single cells, $CD19^{+}$ B cells are selected. On ‘‘not $CD19^{+}$’’ cells, $CD3\varepsilon^{+}$ T cells and $CD11b^{+}$ myeloid cells are selected. $CD3\varepsilon^{+}$ T cells are divided into $CD3\varepsilon^{+}CD4^{+}$ and $CD3\varepsilon^{+}CD8^{+}$ T cells, and $CD11b^{+}$ cells are divided into $CD11b^{+}GR1^{-}$ cells, $CD11b^{+}GR1^{lo}$ cells and $CD11b^{+}GR1^{hi}$ cells. **(C)** Peripheral blood gating strategy. Within live single cells, $SSC\text{-}A^{hi}GR1^{hi}$ cells are selected. On ‘‘not $GR1^{hi}$’’, $CD19^{+}$ B cells and $CD3\varepsilon^{+}$ T cells are selected. $CD3\varepsilon^{-}GR1^{int}$ monocytes are also selected within ‘‘not $GR1^{hi}$’’ cells.

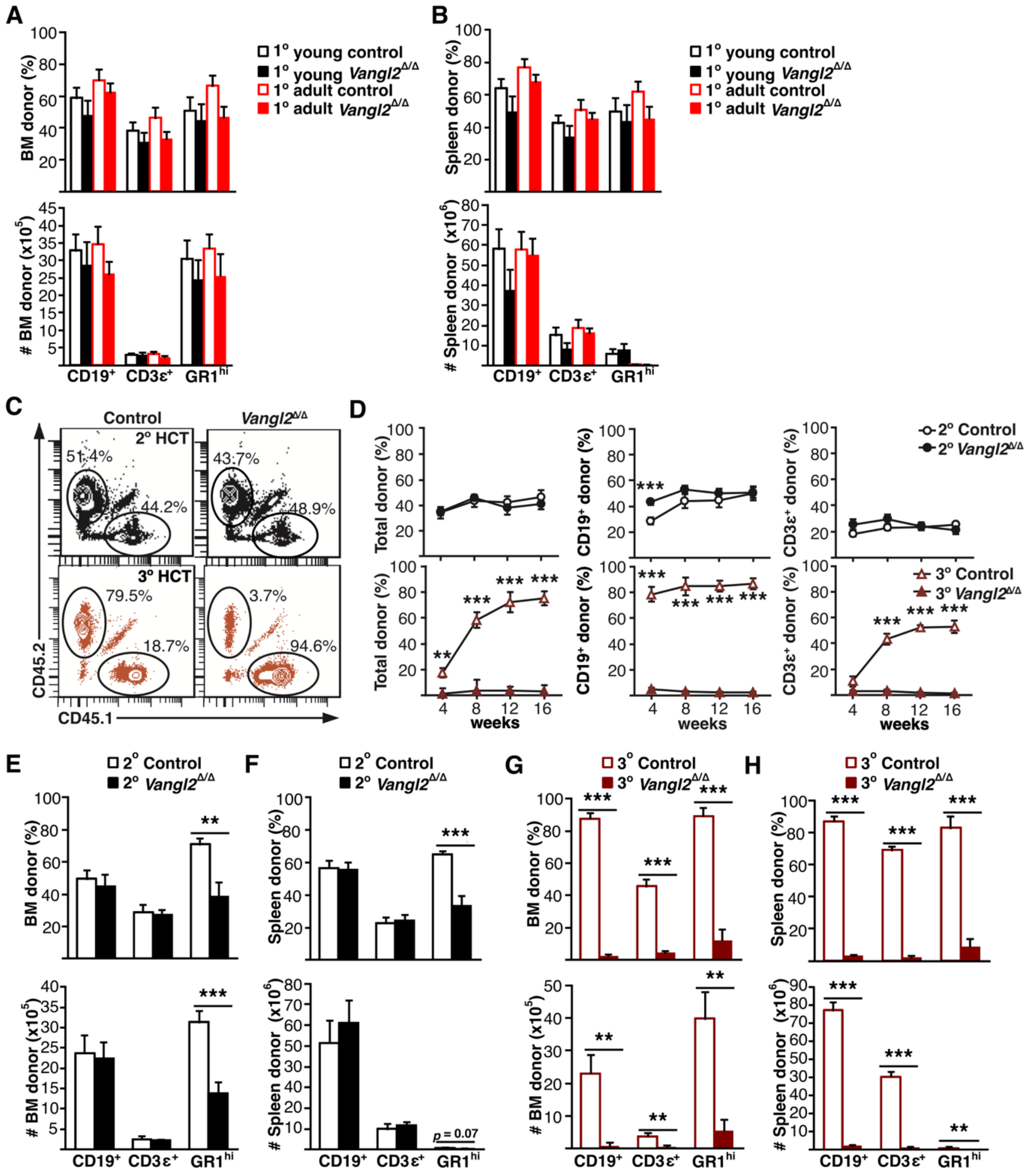

Supplementary Figure 3. Vangl2 loss impairs myeloid cell reconstitution in secondary female recipients, and both myeloid and lymphoid cell reconstitution in tertiary female recipients. Related to Figures 4 and 5.

(A and B) Histograms represent percentage (top) or absolute numbers (bottom) of donor-derived CD19$^+$ B cells, CD3ε$^+$ T cells and GR1^{hi} cells in primary recipients **(A)** BM and **(B)** spleen for young and adult donors (mean + SEM). Young female transplants represent three independent experiments (n=14-16 control, 13-15 *Vangl2*$^{Δ/Δ}$) and adult female transplants represent two independent experiments (n=10 control, 11 *Vangl2*$^{Δ/Δ}$). **(C)** Representative flow cytometry data showing the percentage of donor-derived cells in total secondary and tertiary recipient peripheral blood leukocytes 16 weeks post-transplant. **(D)** Graphs represents pooled results of donor-derived leukocytes, CD19$^+$ B

cells and CD3ε+ T cells in secondary and tertiary recipient peripheral blood (mean + SEM). **(E-H)** Histograms represent percentage (top) or absolute numbers (bottom) of donor-derived CD19+ B cells, CD3ε+ T cells and GR1hi cells in secondary recipients **(E)** BM and **(F)** spleen**,** and in tertiary recipients **(G)** BM and **(H)** spleen (mean + SEM). Secondary female transplants represent two independent experiments (n=9 control, 10 *Vangl2*$^{\Delta/\Delta}$) and tertiary female transplants represent one independent experiment (n=4 control, 6 *Vangl2*$^{\Delta/\Delta}$). *$p \leq 0.05$, **$p \leq 0.005$, ***$p \leq 0.0005$.

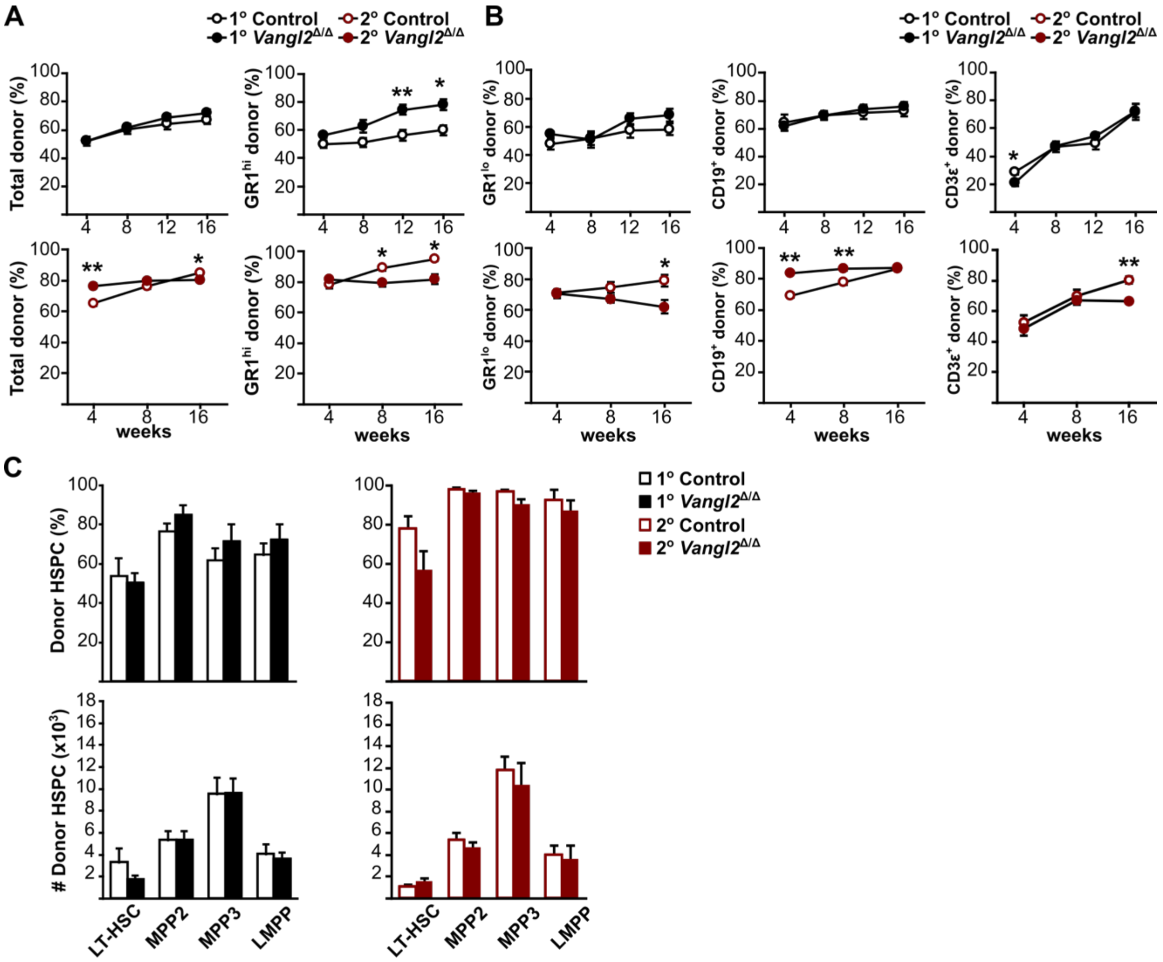

Supplementary Figure 4. Vangl2 loss alters long-term post-transplant peripheral blood reconstitution in secondary male recipients. Related to Figures 4 and 5.

(A) Graphs represent pooled results of donor-derived total and GR1hi cells in male primary (in black) and secondary (in red) recipient peripheral blood (mean + SEM). **(B)** Graphs represent pooled results of donor-derived GR1lo cells, CD19+ B cells and, CD3ε+ T cells in male primary (in black) and secondary (in red) recipient peripheral blood (mean + SEM). **(C)** Histograms represent mean of donor-derived HSPCs percentage or absolute cell numbers in primary and secondary male recipient BM 20 weeks post-transplant (mean + SEM). Primary male transplants represent three independent experiments (n=10 control, 14 *Vangl2*$^{\Delta/\Delta}$), except peripheral blood at 12-16 weeks (two independent experiments (n=7 control, 10 *Vangl2*$^{\Delta/\Delta}$)), and secondary male transplants represent one independent experiment (n=4 control, 5 *Vangl2*$^{\Delta/\Delta}$). No fresh competitor cells were added in male secondary recipients. *$p \leq 0.05$, **$p \leq 0.005$.

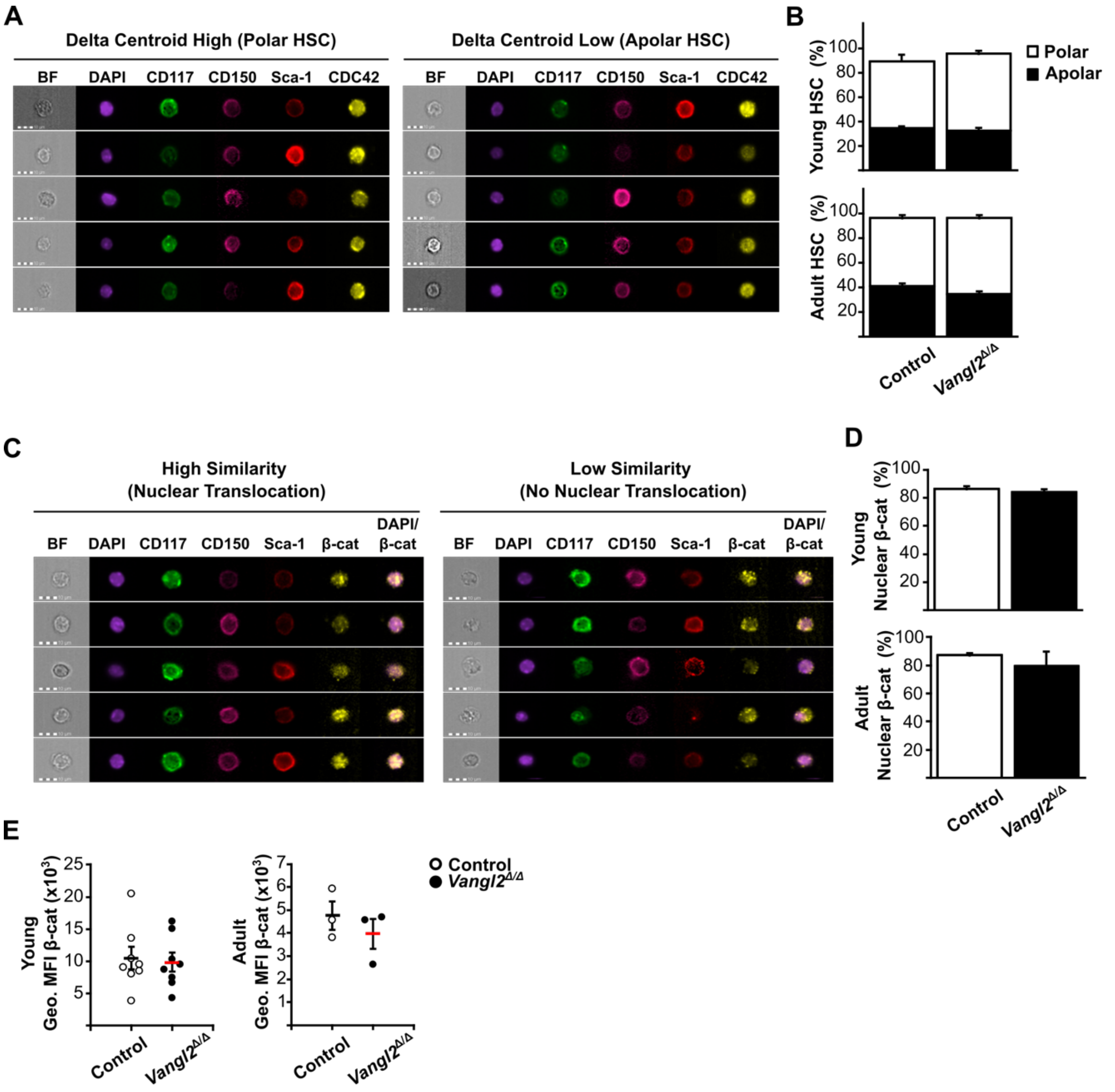

Supplementary Figure 5. Vangl2 loss does not impair Cdc42 localization and β-catenin activation in young and adult mice. Related to Figure 6.

(A) Representative imaging flow cytometry images of polar and apolar $CD150^{+}$ LSK^{+} cells. **(B)** Histograms represents polar and apolar young and adult $CD150^{+}$ LSK^{+} cells (mean + SEM). **(C)** Representative imaging flow cytometry images of β-catenin (β-cat) nuclear translocation based on β-catenin/DAPI similarity analysis. **(D)** Histograms represent mean of nuclear β-catenin localization in young and adult $CD150^{+}$ LSK^{+} cells (mean + SEM). **(E)** β-catenin geometric mean of fluorescence intensity (Geo. MFI) in young and adult $CD150^{+}$ LSK^{+} cells. Young (n=8-10 control, 8-11 *Vangl2*$^{\Delta/\Delta}$) and adult (n=3-4 control, 3-4 *Vangl2*$^{\Delta/\Delta}$) mice represent three independent experiments.
